# Prevalence of Self‐Assessed Masticatory Disorders in Community‐Dwelling Older Adults: A Systematic Review With Meta‐Analysis

**DOI:** 10.1111/joor.14000

**Published:** 2025-05-09

**Authors:** Ilíada Lima Franco, Letícia de Carvalho Palhano Travassos, Renata Veiga Andersen Cavalcanti, Cristiano Miranda de Araujo, Karinna Verissimo Meira Taveira, Leandro Pernambuco

**Affiliations:** ^1^ Programa Associado de Pós‐Graduação em Fonoaudiologia João Pessoa Brazil; ^2^ Programa de Pós‐Graduação em Modelos de Decisão e Saúde João Pessoa Brazil; ^3^ Departamento de Fonoaudiologia Universidade Federal do Rio Grande do Norte Natal Brazil; ^4^ Programa de Pós‐Graduação em Saúde da Comunicação Humana, Universidade Tuiuti do Paraná Curitiba Brazil; ^5^ Programa Associado de Pós‐Graduação em Fonoaudiologia, Universidade Federal do Rio Grande do Norte Natal Brazil; ^6^ Programa de Pós‐Graduação em Saúde da Comunicação Humana, Universidade Federal de Pernambuco Recife Brazil

**Keywords:** aged, elderly, mastication, masticatory disorders, prevalence, systematic review

## Abstract

**Background:**

Aging leads to changes that affect the functionality of the stomatognathic system, which can result in masticatory disorders. The loss or reduction in masticatory efficiency is often reported as one of the main complaints among healthy older adults. Due to variability and imprecision in prevalence estimates, there is a gap in specific knowledge about the true severity of masticatory issues in this population.

**Objective:**

To determine the prevalence of masticatory disorders in community‐dwelling older adults.

**Methods:**

The prevalence of self‐assessed masticatory disorders in community‐dwelling older adults was investigated. A search was conducted in the electronic databases Cinahl, Embase, Lilacs, Livivo, PubMed/Medline, Scopus, Web of Science, Google Scholar, OpenGrey and Proquest. The search strategy was adapted for each database using specific terms and keywords. Population‐based cross‐sectional/ecological studies that used questionnaires to identify masticatory disorders in individuals aged 60 years or older, living in the community were included. Of the 7008 articles identified in the databases and grey literature, 22 articles were included for data extraction and analysis.

**Results:**

High heterogeneity was observed among the prevalence estimates (*I* ^2^ = 100%) for the different studies included in the analysis, which was not explained by the mean age of the study population or sample size when evaluated using a meta‐regression model (*p* < 0.05). The pooled prevalence of masticatory disorders was 36% (95% CI = 0.28–0.43; *I* ^2^ = 100%), with individual study estimates ranging from 4.3% to 61.7%.

**Conclusion:**

The prevalence of self‐assessed masticatory disorders in community‐dwelling older adults is approximately 36%.

## Graphical abstract

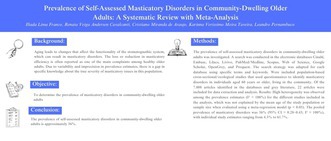

## 1 Introduction

Aging is a natural, gradual, global and inherent process that leads to changes in anatomy and physiology, affecting the stomatognathic system [1, 2]. These age‐related changes often result in a decline in masticatory function, such as reduced efficiency in food bolus preparation, difficulty in grinding and pulverising food and decreased bite force. These masticatory changes are particularly important to assess in older adults, as they can impact overall health and quality of life [3].

However, measuring masticatory disorders presents significant challenges due to the complex nature of the stomatognathic system and the various factors that influence its function, such as dental status, prosthetic adaptation and oral health conditions [4, 5]. Loss or reduction in masticatory efficiency is frequently cited as one of the main complaints among healthy older adults [6].

Previous studies indicate that the prevalence of unsatisfactory masticatory capacity in community‐dwelling older adults ranges from 14.3% to 49.7% [7, 8, 9]. However, there is considerable variability in the methods used to assess masticatory disorders across studies, with some relying on subjective self‐reports, while others use clinical evaluations. This lack of standardisation complicates the comparison of prevalence estimates and the understanding of the true impact of masticatory issues in the elderly population.

Given the complexities involved in accurately measuring masticatory disorders, it is essential to better define and address these parameters in research. It is crucial to assess the prevalence of masticatory disorders in community‐dwelling older adults to develop effective strategies to promote and prevent declines in masticatory capacity [10]. Therefore, the aim of this systematic review and meta‐analysis is to determine the prevalence of masticatory disorders in community‐dwelling older adults and to highlight the challenges in assessing these conditions.

## 2 Methods

This systematic review was conducted in accordance with the *Preferred Reporting Items for Systematic Reviews and Meta‐Analyses* (PRISMA) guidelines [11]. The review protocol was registered in the International Prospective Register of Systematic Reviews (PROSPERO) (CRD42020202495).

### 2.1 Search Strategy and Selection Criteria

The search was conducted in the following databases: Cinahl, Embase, Lilacs, Livivo, PubMed/Medline, Scopus, Web of Science, and in grey literature sources such as Google Scholar, Open Grey and Proquest, including theses and dissertations. The search strategy was tailored to each database, and the terms were selected from the PubMed MeSH terms and EMBASE EMTREE Terms, considering the studied condition, exposure and outcomes included in the review.

References were managed, and duplicates were removed using virtual software (EndNote Web). The articles were selected through two phases, both conducted independently by the same reviewers (I.L.F. and L.C.P.T.). In Phase 1, articles were selected based on the title and abstract, excluding those that did not meet the inclusion criteria. In Phase 2, full texts were read by the same reviewers, and studies were excluded according to eligibility criteria.

Eligible studies included original articles reporting the prevalence of masticatory disorders in community‐dwelling older adults of both sexes, aged 60 years or older, where the method or sampling process clearly indicated that the population was representative of the source population. Studies using self‐reported symptom questionnaires, whether validated or not, to determine the prevalence of masticatory disorders were included. The search included studies in any language and without restrictions on publication time.

Studies were excluded if they included individuals under 60 years of age, children, adolescents and adults alongside individuals aged 60 years or older, and if they did not present age‐stratified data in the results. Also excluded were studies that did not report prevalence results for masticatory disorders or did not provide sufficient data to calculate estimates, studies that assessed masticatory efficiency or performance using colorimetric capsules, colour‐changing chewing gum, gum tests, sieving systems or masticatory function with food. Studies exclusively investigating residents of nursing homes, individuals receiving home care or those with specific diseases or undergoing dental prosthesis evaluations were also excluded, as were reviews, letters, books, conference abstracts, case reports, case series, opinion articles, technical papers, guidelines and studies without population‐based samples.

Data from the selected articles were tabulated according to characteristics such as author, year of publication, country of publication, sample size, mean age of participants, percentage of men and women, reported prevalence and the method of assessment used.

### 2.2 Risk of Bias Assessment

Each study was analysed individually and independently by two reviewers (I.L.F. and L.C.P.T.). In cases of disagreement between the investigators, a third reviewer (R.C.) was consulted. The risk of bias was assessed using the *Joanna Briggs Institute's critical appraisal tools: Checklist for Cross‐Sectional Studies* [12].

### 2.3 Meta‐Analysis

A meta‐analysis of proportions was conducted using a random‐effects model, weighted by the inverse of the variance. For the calculation of variance, represented by the Tau values, the DerSimonian–Laird estimator was used [13]. Heterogeneity between studies was assessed using the inconsistency index (*I* ^2^) [14]. Confidence intervals of 95% were calculated using the Clopper–Pearson method. All analysis and the construction of the forest plot were performed in the RStudio programming environment, version 1.2.1335 (RStudio Inc., Boston, USA), using the R programming language.

To ensure greater robustness of the estimates obtained, a sensitivity analysis was planned to check whether any study with low statistical power could have distorted the results. For this, a sample calculation was conducted considering margins of error of 5% and 1%, with 95% confidence intervals. The sample size was based on the prevalence obtained from the inclusion of all studies, assuming inference for an infinite population. A random‐effects meta‐regression was also performed to assess the influence of the mean age of the studies included in the analysis on the variation of the observed effect size, with a significance level of 5%.

In order to identify any possible source of heterogeneity, a subgroup analysis was conducted, dividing the studies into four groups based on the definitions used to determine masticatory disorders (‘chewing difficulty’, ‘chewing disability’, ‘chewing problem’ and ‘chewing discomfort’). In this regard, an extensive search was also conducted, including a database in a language other than English (LILACS), which reduced the likelihood of this bias. Additionally, a publication bias was assessed by funnel plot and Egger's test.

## 3 Results

### 3.1 Study Selection

**FIGURE 1:**
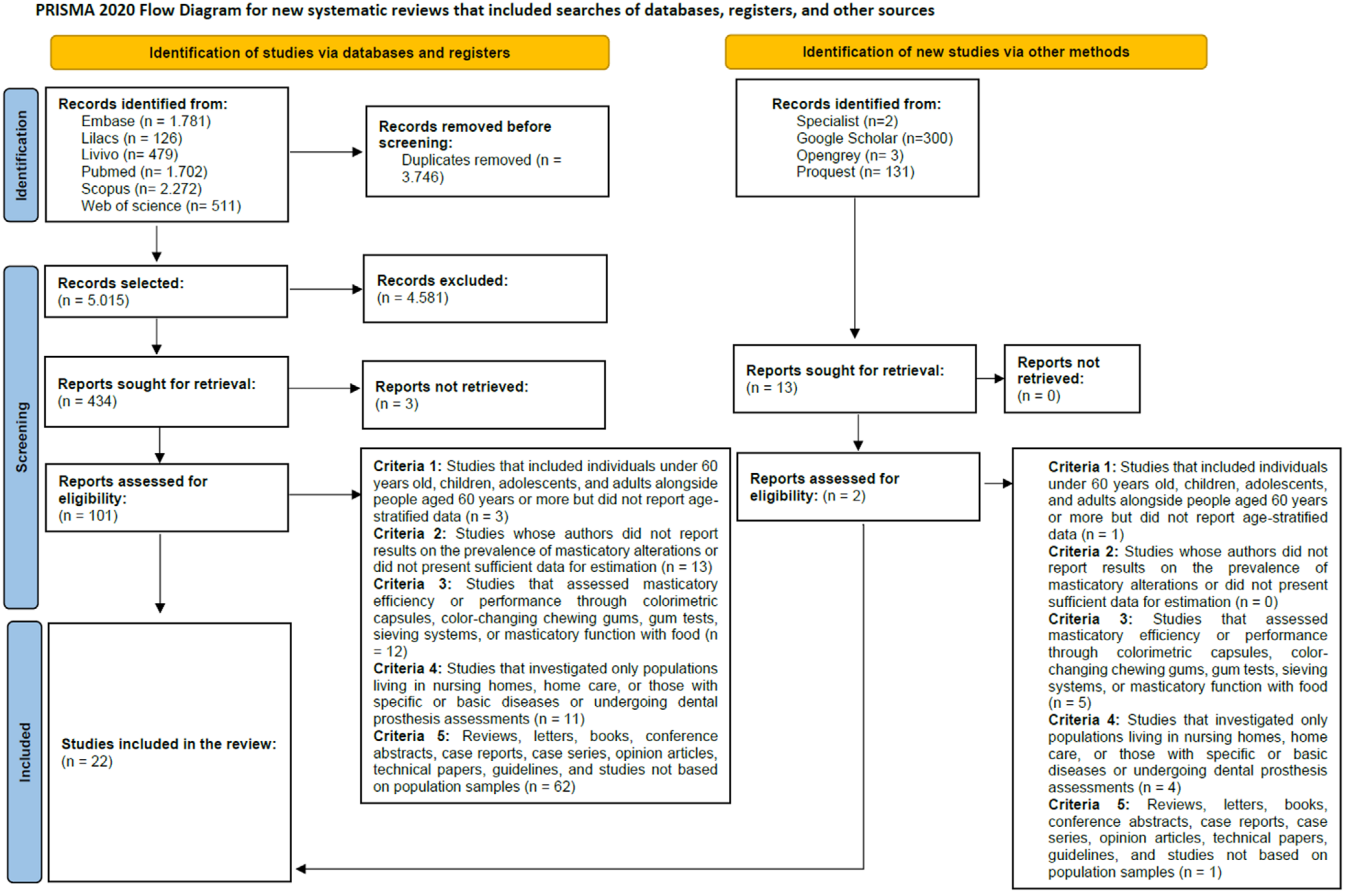
Flowchart of the search and selection phases of the systematic review. *Source:* Page et al. [15].

Figure 1 shows the flowchart describing the study identification process. A total of 8761 articles were found. After removing duplicates, 5015 studies remained for the step of carefully analysing titles and abstracts, resulting in 434 studies for the next phase. After reading the full texts, 30 articles were retained for data extraction, with 2 additional studies selected by the expert on the review team. In the end, 22 studies were included for result synthesis.

### 3.2 Characteristics of the Studies

Of the 22 selected articles, 14 were from Asian countries. The sample size ranged from 557 [16] participants to 63 602 [17]. The reported prevalence varied from 4.3% [18] of individuals dissatisfied with bite and chewing to 61.7% [19] of individuals reporting discomfort while chewing. A detailed summary of the characteristics of the included studies, as well as their main results, is presented in Table 1.

**TABLE 1:** Characteristics of the included studies and their main results.

Author, year, country	Sample (*n*)	Mean age (SD) and/or min–max	Gender (% female/% male)	Questionnaire or question and response options	Outcome representing chewing change	Prevalence and location
Aakriti et al. (2021); India [20]	1003	69.5 years (male) and 67.8 years (female)	63.8%/36.9%	Not reported	Chewing problem	34.3% District Nainital, Uttarakhand state, India
Cho and Kim (2019); Korea [21]	3034	Not reported (age 65 and older)	According to chewing ability: Poor: 59.9%/40.1% Intermediate: 59.3%/40.7% High: 53.8%/46.2%	Question: Was assessed on a 5‐point scale using the question ‘Do you feel uncomfortable with chewing food due to tooth, denture, gum disease or other oral problems?’ Answer: Chewing ability was classified into three groups: ‘low’ group—participants' responses were ‘very uncomfortable’ and ‘uncomfortable’; ‘middle’ group responses were ‘fair’; and ‘high’ group—responses were ‘comfortable’ and ‘very comfortable’	Chewing ability classified as low, intermediate or high	Poor: Ages 65–69: 33.1% Ages 70–74: 29.8% Ages 75 and older: 37.1% Intermediate: Ages 65–69: 38.5% Ages 70–74: 32.1% Ages 75 and older: 29.3% High: Ages 65–69: 41.3% Ages 70–74: 27.1% Ages 75 and older: 31.7% Korea
Dias‐da‐Costa et al. (2010); Brazil [22]	5124	67–74 years	61.0%/39.0%	It was categorised by chewing ability Answer: Excellent/good or fair/poor/very poor (the latter classified as ‘unsatisfactory’)	Chewing disability	49.7% Urban and rural areas of 250 municipalities across all Brazilian states
Kamdem et al. (2017); Switzerland [22, 23]	992	74.9 years	59.8%/40.2%	Question: ‘Are you able to chew all types of food?’ Answer: ‘Yes, but hardly’ or ‘no, I swallow whole’ vs. ‘yes, without difficulty’	Chewing disability	9.7% Lausanne, Switzerland
Kida et al. (2007); Norway [24]	1031	62.9 years	53.6%/46.4%	Not reported Answer: Inability to chew all foods or soft/puree only	Inability to chew all foods	30% Pwani, Eastern Tanzania, and the capital Dar es Salaam
Kim et al. (2023); Korea [25]	3437	Not reported (age 65 and older)	58.3%/41.7%	Question: Chewing discomfort was assessed with a self‐administered questionnaire Answer: If participants checked ‘very uncomfortable’, ‘inconvenient’ or ‘just so’, classified it into ‘chewing discomfort’ and if participants checked ‘not uncomfortable’ and ‘not at all uncomfortable’, classified as ‘no chewing discomfort’	Chewing discomfort	37.1% Korea
Kim and Jin (2018); Korea [26]	2904	Ages 65–74: 65.6% and ages 75–84: 31.4%	43.0%/56.9%	Not reported Answer: Chewing problems were classified into three categories: good, ordinary and bad	Chewing problems	61.6% Korea
Laudisio et al. (2016); Italy [27]	1155	Chewing difficulty: 77.7 years and no chewing difficulty: 74.2 years	Chewing dysfunction: 63%/37% and no chewing dysfunction: 53%/47%	Question: ‘Do you experience difficulty chewing?’ and ‘Has the amount of food you usually eat decreased in the last year because of chewing problems?’ Answer: Yes or no (was diagnosed if either of these questions was answered affirmatively)	Chewing difficulty and chewing problems	35.06% Tuscany, Italy
Lee et al. (2015); Korea [28]	9840	Ages 65–74: 36.3%; ages 75–84: 42.3%; ages 85 and older: 46.7%	15.2%/24.8%	Question: ‘Have you developed difficulty in chewing food over the past 6 months?’ Answer: Yes or no	Difficulty chewing in the past 6 months	26.3% Korea
Lexoboon et al. (2012); Sweden [16]	557	83.0 years	58.9%/41.1%	Question: ‘Can you chew hard food such as hard bread or apples?’ Answer: ‘Yes, without difficulty’ was classified as not having chewing difficulty. The answers ‘Yes, but I must be careful’ and ‘No, not at all’ were classified as having chewing difficulty	Chewing difficulty	20.8% Stockholm, Sweden
Lo et al. (2016); Taiwan [9]	1793	Ages 65–69: 38.6%; ages 70–74: 33.4%; ages 75–79: 17.8%; over 80 years: 10.3%	49.6%/50.3%	Question: ‘Do you have difficulty chewing food?’ Answer: Yes or no	Chewing difficulty	37.3% Hakka, mountainous areas of Eastern, Penghu, Northern, Central and Southern Taiwan
Milagres et al. (2018); Brazil [29]	2126	70.91 years in younger elderly and 82.70 years in older elderly	65.6%/34.4%	Question: Difficulty chewing and swallowing food and difficulty or pain chewing hard foods Answer: Yes or no	Difficulty or pain chewing hard foods	36.8% Cities in Brazil: Campinas (SP), Belém (PA), Parnaíba (PI), Poços de Caldas (MG), subdistrict of Ermelino Matarazzo in São Paulo (SP) and Ivotí (RS)
Milagres et al. (2022); Brazil [30]	2341	72.3 years (± 5.48)	65.6%/34.4%	Question: Difficulty chewing and swallowing food and difficulty or pain chewing hard foods Answer: Yes or no	Difficulty or pain chewing hard foods	35.5% Cities in Brazil: Campinas (SP), Belém (PA), Parnaíba (PI), Poços de Caldas (MG), subdistrict of Ermelino Matarazzo in São Paulo (SP) and Ivotí (RS)
Moon and Hong (2017); Korea [19]	1126	72.3 years	56.5%/43.5%	Question: ‘Are you experiencing discomfort when chewing food due to problems in your mouth, such as teeth, dentures or gums?’ Answer: 5‐point scale from: very inconvenient to not at all uncomfortable	Chewing discomfort	61.7% Korea
Nascimento et al. (2024); Singapore [31]	973	69.3 years (± 6.52)	52.1%/47.9%	Question: Participant self‐rating based on their correspondence with the toughness values of the food items in the Japanese list, a food item group list tailored to the Singaporean context was arranged in descending order of food toughness Answer: It was considered chewing disability as the inability to bite and chew the top two toughest food item groups	Chewing disability	33% Singapore
Park et al. (2013); Korea [32]	4924	Not reported (age 65 and older)	58.6%/41.4%	Question: ‘Do you experience discomfort when chewing food?’ Answer: Those who answered ‘very uncomfortable’ or ‘uncomfortable’ were classified as having chewing difficulty. Those who answered ‘somewhat’, ‘not uncomfortable’ or ‘not at all uncomfortable’ were classified as not having chewing difficulty.	Chewing discomfort	54.3% Korea
Park and Son Hong (2017); Korea [33]	10 543	74.29 years (± 5.91)	59.1%/40.9%	Question: Self‐report of chewing ability representing levels of comfort when chewing meat or solid food Answer: ‘Very good’ or ‘good’ were recoded as ‘good’, and ‘poor’ or ‘very poor’ were recoded as ‘poor’	Poor chewing ability	56.9% Korea
Shin et al. (2022); Korea [34]	3076	No chewing discomfort: 70.9 years (± 0.12) With chewing discomfort: 71.6 years (± 0.22)	54.6%/45.4%	Question: ‘Do you feel uncomfortable chewing food due to mouth problems, such as teeth, dentures or gums? (If you have a denture, please tell us how you feel.)’ Answer: ‘very much’, ‘quite a lot’, ‘some’, ‘very little’, or ‘not at all’. Chewing discomfort was categorised into two groups for the analysis: yes (very much, quite a lot) and no (some, very little, not at all)	Chewing discomfort	43.3% Korea
Shiota et al. (2023); Japan [17]	63 602	73 years (± 5.5)	52.0%/48.0%	Question: ‘Do you have any difficulties eating tough foods compared to 6 months ago?’ Answer: Yes or no	Chewing difficulty	23.9% Japão
Srinarupat et al. (2022); Tokyo [35]	2310	67.2 years	50.2%/49.8%	Question: ‘Do you have chewing problems?’ Answer: ‘No’ for those who answered ‘no chewing problems’ and ‘yes’ for those who answered either ‘sometimes, but I can chew’ or ‘yes, I have severe chewing problems’	Chewing problems	53.3% Thailand
Steele et al. (1997); England [18]	2280	68.1 years	51.7%/48.3%	Not reported	Dissatisfaction with biting and chewing ability	4.3% Three geographically distinct areas of England
Yamamoto‐Kuramoto et al. (2023), Japan [36]	44 083	73.7 years	53.2%/46.8%	Question: ‘Do you find it more difficult to eat hard food than you did 6 months ago?’ Answer: Yes or no	Chewing difficulty	27.1% Nine municipalities in Japan

### 3.3 Assessment of Risk of Bias

**FIGURE 2:**
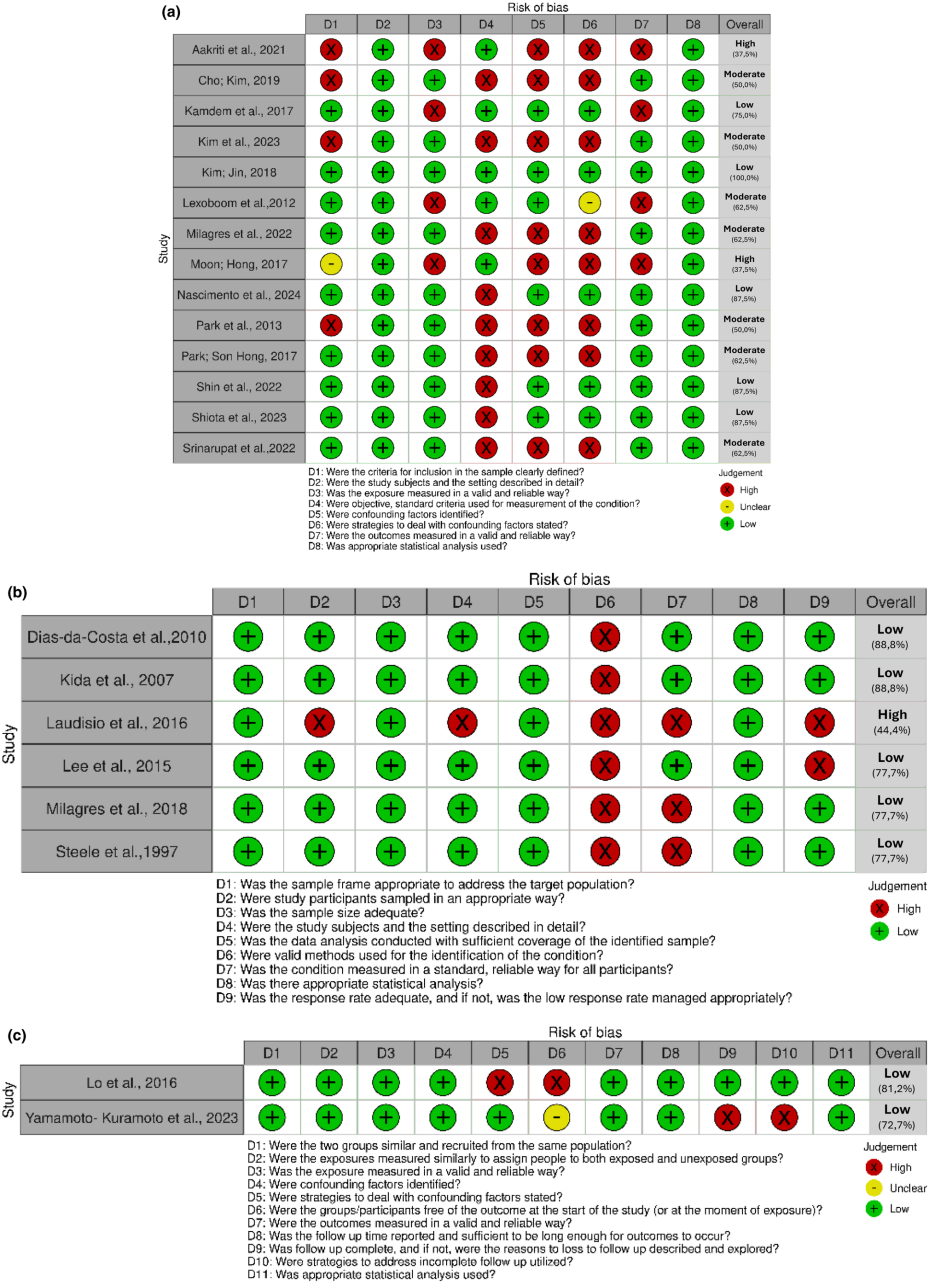
Risk of bias assessment for (a) cross‐sectional studies, (b) prevalence studies and (c) cohort studies.

Regarding the overall risk of bias, within the studies classified as cross‐sectional, two studies were classified as high risk of bias, seven were classified as moderate risk of bias and five were classified as low risk of bias. Among the studies classified as prevalence studies, five were classified as low risk of bias and one was classified as high risk of bias. In the cohort studies, both were classified as low risk of bias. The results on the risk of bias are described in Figure 2 (Figure 2A for cross‐sectional studies; Figure 2B for prevalence studies; Figure 2C for cohort studies).

### 3.4 Meta‐Analysis

For the meta‐analysis, 21 of the 22 studies identified were considered; the study by Cho and Kim [21], was excluded from the analysis as it reported different prevalences of masticatory disorders based on age group and the level of those alterations.

**FIGURE 3:**
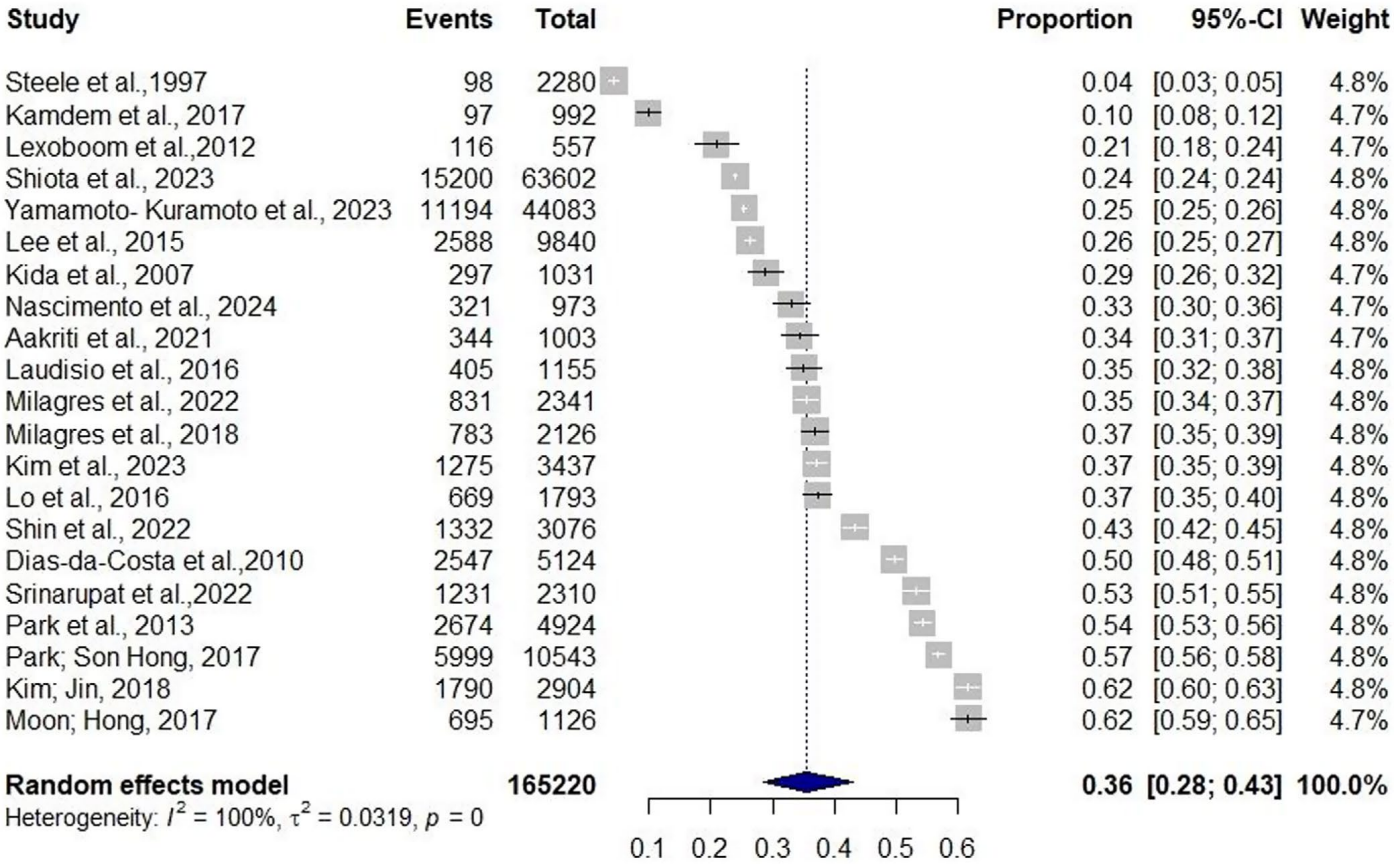
Forest plot of the meta‐analysis of the prevalence of chewing alterations in community‐dwelling elderly individuals.

A total of 165 220 community‐dwelling elderly individuals were included in the analysis from the 21 selected studies. The pooled prevalence of masticatory disorders was 36% (95% CI = 28%–43%; *I* ^2^ = 100%) among the elderly (Figure 3). The average age of the samples in the included studies did not appear to influence the observed prevalence when analysed using a meta‐regression model (*p* > 0.05).

In the sensitivity analysis based on sample size calculation with a 5% margin of error, the required sample size was 354 individuals, allowing all studies to be included in the analysis. However, with a more stringent 1% margin of error, the required sample size increased to 8774 individuals, resulting in the inclusion of only 4 studies, which slightly changed the estimates to 33% (95% CI = 19%–48%; *I* ^2^ = 100%).

No publication bias was detected by the funnel plot and the Egger's test (*p* = 0.063) (Appendix A). The existing heterogeneity in the analysis persisted even when subdivided into subgroups. The estimated prevalence according to subgroups was 36% (95% CI = 28%–43%; *I* ^2^ = 100%), underlying shown in Appendix B.

## 4 Discussion

The notable increase in interest in understanding the chewing process, along with questions about how this process is affected over the years in individuals, has created a demand to understand and know the prevalence of these alterations in community‐dwelling elderly individuals. Most of the studies published were from the period between 2007 and 2024, with the majority conducted in the last 5 years.

The findings indicate significant variation in the locations where the studies were conducted. Additionally, there was variation in the geographical perspective, with data collection sites ranging from districts, states, municipalities, cities, sub‐districts and villages. This geographical and territorial diversity led to substantial inconsistency in sample sizes across the selected studies, ranging from 557 [16] individuals in a city in Sweden to 63 602 [17] participants in Japan.

The percentage of age and sex was homogeneous among the selected studies, with 86.36% of the articles having more females in their samples than males. This result may relate to the general observation that women use healthcare services more frequently than men, possibly due to greater interest in their health and a higher willingness to participate in research and surveys.

Another point for discussion is the significant divergence in data collection methods among the selected articles. Although all used self‐assessment methods, the questions posed by researchers to collect the data of interest differed in each study.

Although self‐reported questionnaires were primarily developed for epidemiological purposes, as in this study, and are frequently used due to their practicality and relatively low cost, the predominance of self‐reported data in many of the included studies still represents a significant limitation. This occurs because they are subject to recall bias and the subjectivity of participants' responses. Individuals may underestimate or overestimate masticatory problems based on their personal perception of oral health, educational level or even the phrasing of survey questions. This subjectivity can compromise the accuracy and comparability of prevalence estimates across studies [37, 38].

Additionally, the absence of objective evaluations in some studies limits the ability to validate self‐reported information. Objective methods, such as clinical assessments conducted by trained professionals or the use of standardised instruments, provide more reliable and consistent estimates. The lack of these evaluations may also obscure conditions that are not perceived or recognised by participants, leading to underreporting of masticatory disorders [37, 38].

These limitations highlight the importance of future studies that combine subjective and objective approaches. Furthermore, guidelines recommend using specific self‐reported questionnaires, and opting to use them can improve data accuracy and contribute to a more robust understanding of the prevalence and impact of masticatory disorders in the elderly population [39].

Despite this, articles could be subdivided into groups based on the definitions used to determine masticatory disorders. The first group used the term ‘chewing difficulty’ [9, 16, 17, 27, 28, 29, 30, 36], the second group defined it as ‘chewing disability’ [19, 21, 22, 23, 24, 33], the third group referred to it as ‘chewing problem’ [20, 26, 27, 35], and the fourth group defined it as ‘chewing discomfort’ [19, 25, 32, 34].

However, some articles did not fit into any of these groups and used concepts like ‘chewing dissatisfaction’ [18]. This variability in terminology reflects the lack of standardisation in obtaining these data.

Only one of the selected articles provided a definition of what characterised chewing dysfunction, which was reported symptoms or an objective deficit in chewing selected foods [27]. Other articles only specified the question used to obtain the data, and in some cases, this information was omitted. This created difficulties in data tabulation, as it was not clear how each study conceptualised the reported symptoms.

The selected articles showed significant variation in the prevalence rates found, ranging from 4.3% [18] to 61.7% [19]. This variation could not be justified by factors such as age and sex.

However, methodological differences, lack of definition for chewing problems, data collection methods, absence of validity evidence for instruments and lack of a standardised questionnaire model led to high heterogeneity among the selected studies.

A subgroup analysis was conducted, dividing the studies into four groups based on the definitions used to determine masticatory disorders (‘chewing difficulty’, ‘chewing disability’, ‘chewing problem’ and ‘chewing discomfort’) in an attempt to mitigate the high heterogeneity observed. However, the heterogeneity persisted. Systematic reviews of prevalence studies often exhibit high heterogeneity due to variations in sample sizes, population characteristics and methodological differences [40]. These factors likely contributed to the substantial heterogeneity in the study at hand.

To address these challenges, a sensitivity analysis was incorporated to explore how the findings were influenced under different scenarios, such as stricter margins of error or the exclusion of studies with low statistical power. However, although the sensitivity analyses provided insights into the stability of the estimates, they also highlighted the complexity of drawing definitive conclusions when faced with methodological inconsistencies across studies. A more in‐depth discussion of these sensitivity scenarios strengthens the robustness of the study's findings and underscores the need for standardised criteria in future research to reduce heterogeneity and improve interpretability.

In this review, three studies [19, 20, 27] presented a high risk of bias with low methodological quality. This was primarily due to poor methodological descriptions, lack of reliable methods for assessing the prevalence of chewing alterations, resulting in unreliable results, failure to identify confounding factors and lack of strategies to address these factors.

Seven studies [16, 21, 25, 30, 32, 33, 35] were classified as having a moderate risk of bias and methodological quality, mainly due to the lack of objective and standardised criteria for measuring the condition and not identifying and controlling confounding factors.

Ultimately, 12 studies demonstrated a low risk of bias and high methodological quality [9, 17, 18, 22, 23, 24, 26, 28, 29, 31, 34, 36] presented a low risk of bias with high methodological quality. These studies described their methodology in more detail. However, the limitations previously mentioned should be considered.

This is the first systematic review to estimate the prevalence of masticatory disorders in community‐dwelling elderly individuals. Such knowledge can contribute to understanding the magnitude of the number of elderly affected by these alterations and fill this knowledge gap. By discussing these findings within a broader functional health model, we can highlight the profound impact of chewing difficulties on other aspects of health, such as nutrition, mental well‐being and social interaction. This information could encourage studies investigating associated factors and inform public health strategies to address potential consequences like weight loss and food refusal.

However, it is clear that methods need to be implemented to overcome the methodological barriers found in the original studies to produce knowledge with fewer confounding criteria, greater rigour and a higher level of evidence certainty. This study is important not only in raising awareness of masticatory disorders but also in guiding preventive strategies to enhance the overall health and quality of life.

## 5 Conclusion

The pooled prevalence of masticatory disorders in community‐dwelling elderly individuals was 36% (95% CI = 0.28–0.43; *I* ^2^ = 100%).

## Author Contributions

**Ilíada Lima Franco** and **Letícia de Carvalho Palhano Travassos:** conducted the literature search in databases, was responsible for the methodological quality assessment of the included studies, performed data extraction, created tables and graphs to synthesise the information and assisted in manuscript writing. **Renata Veiga Andersen Cavalcanti:** contributed to the interpretation of the results and the development of implications for future research. Also ensured the accuracy of the study's conclusions. **Cristiano Miranda de Araujo:** provided a critical review of the manuscript, conducted statistical analyses, such as meta‐analyses when necessary and provided technical support in using software and tools for data analysis. **Karinna Verissimo Meira Taveira** and **Leandro Pernambuco:** contributed to the development of the analysis methodology, interpretation of results and formulation of implications for future research. Also ensured the accuracy of the study's conclusions.

## Conflicts of Interest

The authors declare no conflicts of interest.

## Peer Review

The peer review history for this article is available at https://www.webofscience.com/api/gateway/wos/peer‐review/10.1111/joor.14000.

## Data Availability

The data that support the findings of this study are available from the corresponding author upon reasonable request.
